# Supplementary material for: Transcriptomic Profiles of CD47 in Breast Tumors Predict Outcome and Are Associated with Immune Activation
Source: Int J Mol Sci. 2021 Apr 7;22(8):3836. doi: 10.3390/ijms22083836 (PMC8067872; doi:10.3390/ijms22083836)
Supplement: Supplementary file 1 [file ijms-22-03836-s001.zip › Supplementary Figure 4.pptx]

## Slide 1
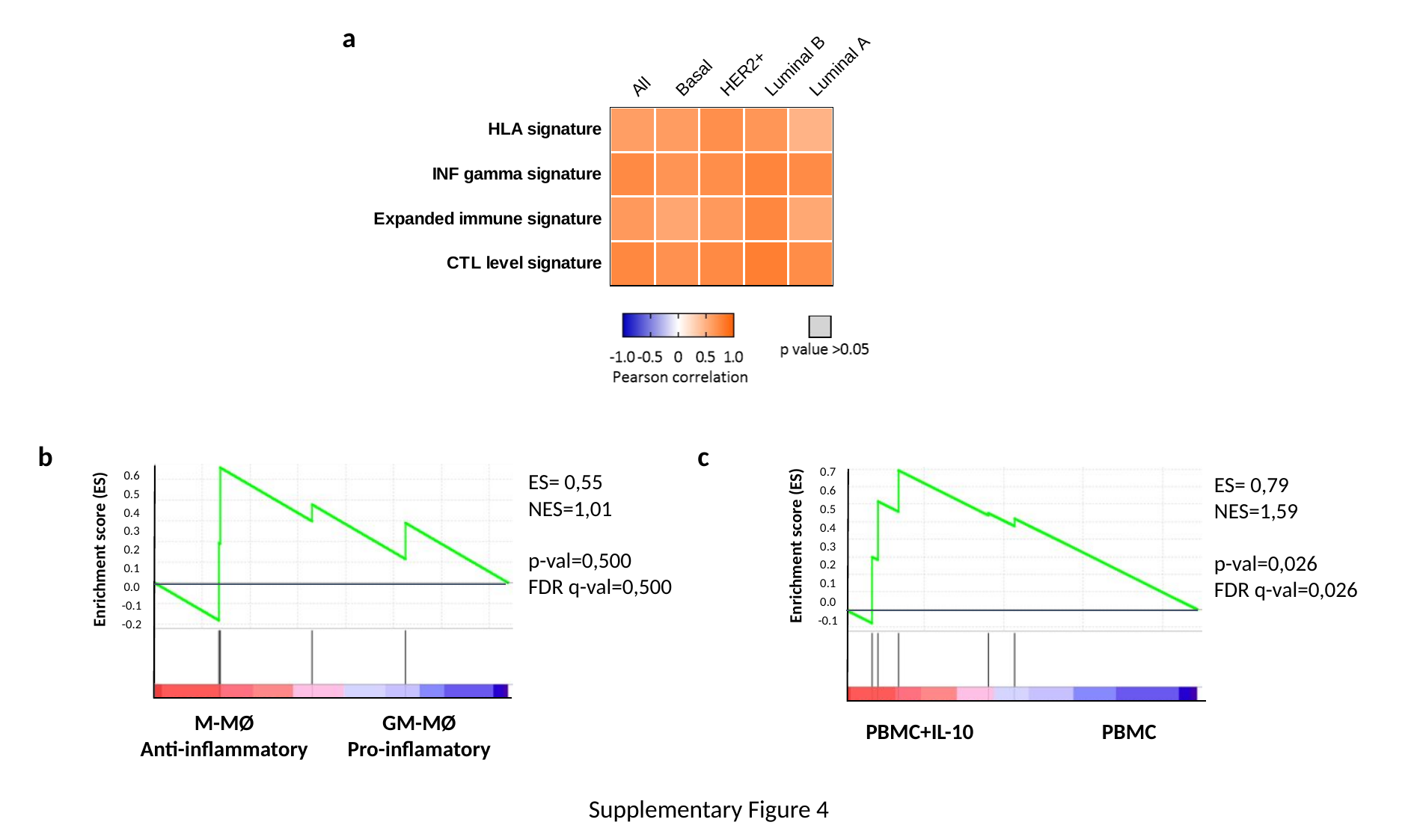

a
b
c
0.7
0.6
0.5
0.4
Enrichment score (ES)
0.3
0.2
0.1
0.0
-0.1
0.6
0.5
0.4
0.3
Enrichment score (ES)
0.2
0.1
0.0
-0.1
-0.2
ES= 0,55
NES=1,01
p-val=0,500
FDR q-val=0,500
M-MØ
Anti-inflammatory
GM-MØ
Pro-inflamatory
ES= 0,79
NES=1,59
p-val=0,026
FDR q-val=0,026
PBMC+IL-10
PBMC
Supplementary Figure 4
