# Supplementary figures and images for: Transcriptomic Profiles of CD47 in Breast Tumors Predict Outcome and Are Associated with Immune Activation
Source: Int J Mol Sci. 2021 Apr 7;22(8):3836. doi: 10.3390/ijms22083836 (PMC8067872; doi:10.3390/ijms22083836)

## Slide 1
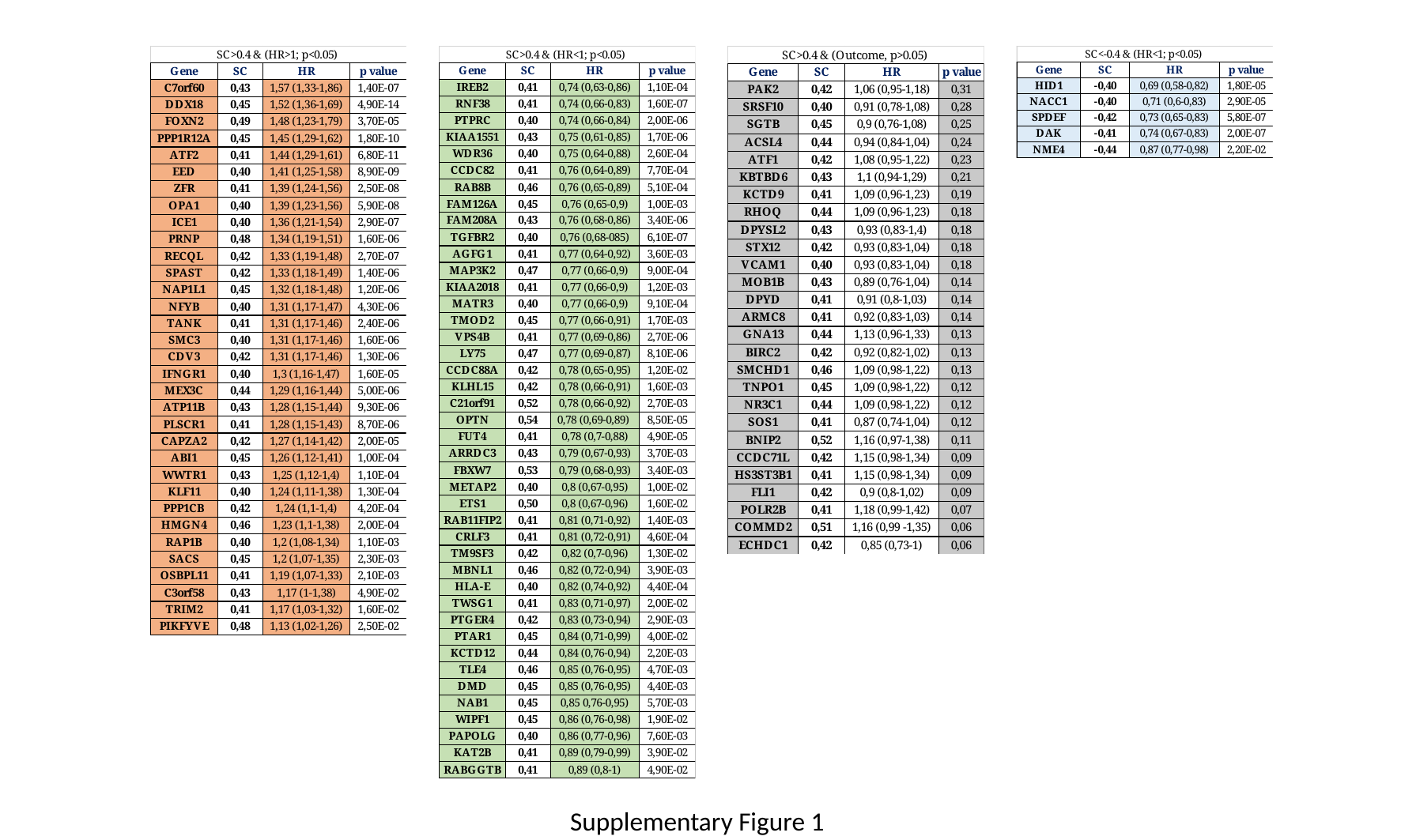

Supplementary Figure 1

Supplement: Supplementary file 1 [file ijms-22-03836-s001.zip › Supplementary Figure 1.pptx]

## Slide 1
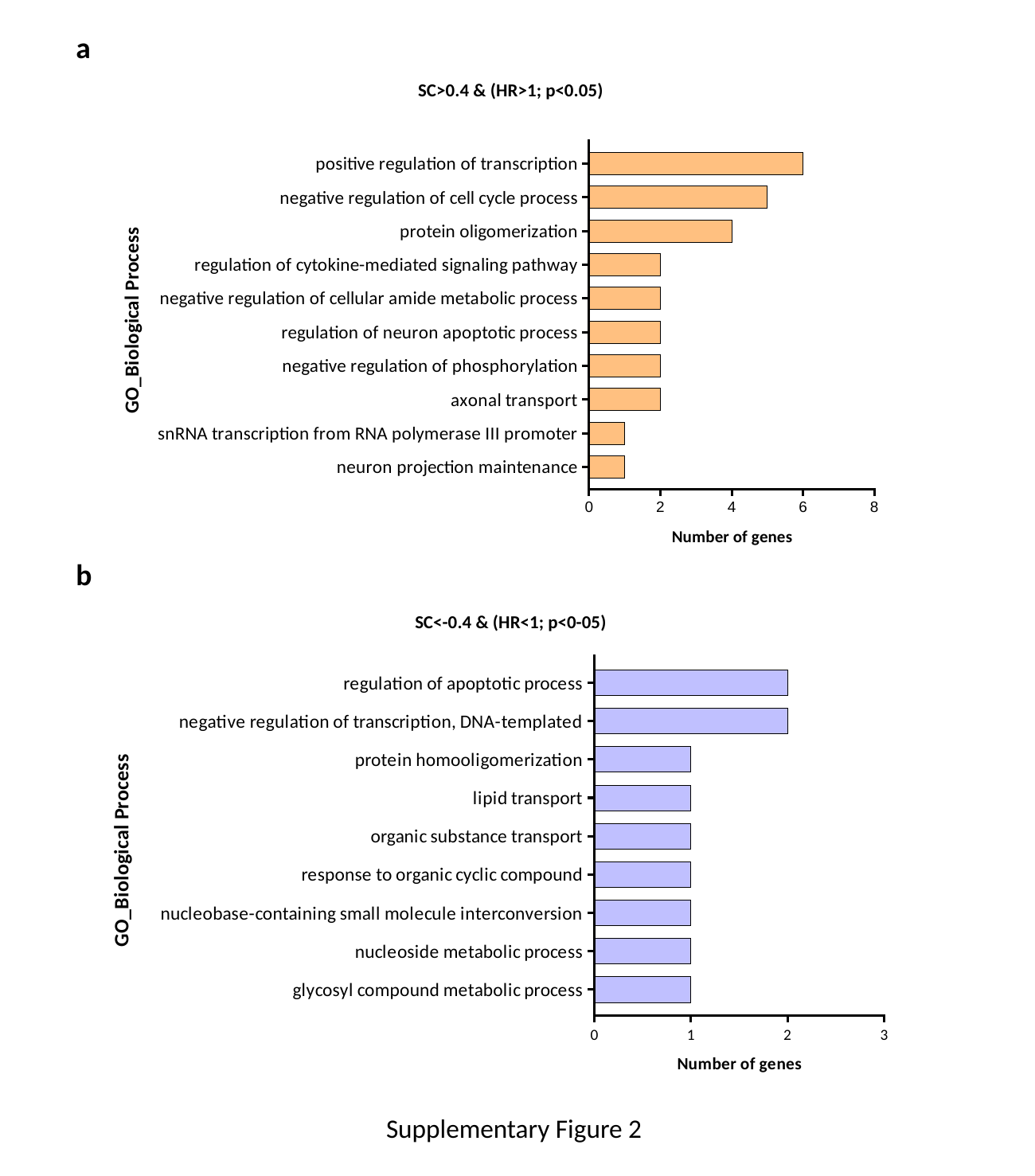

a
SC>0.4 & (HR>1; p<0.05)
b
SC<-0.4 & (HR<1; p<0-05)
Supplementary Figure 2

Supplement: Supplementary file 1 [file ijms-22-03836-s001.zip › Supplementary Figure 2.pptx]

## Slide 1
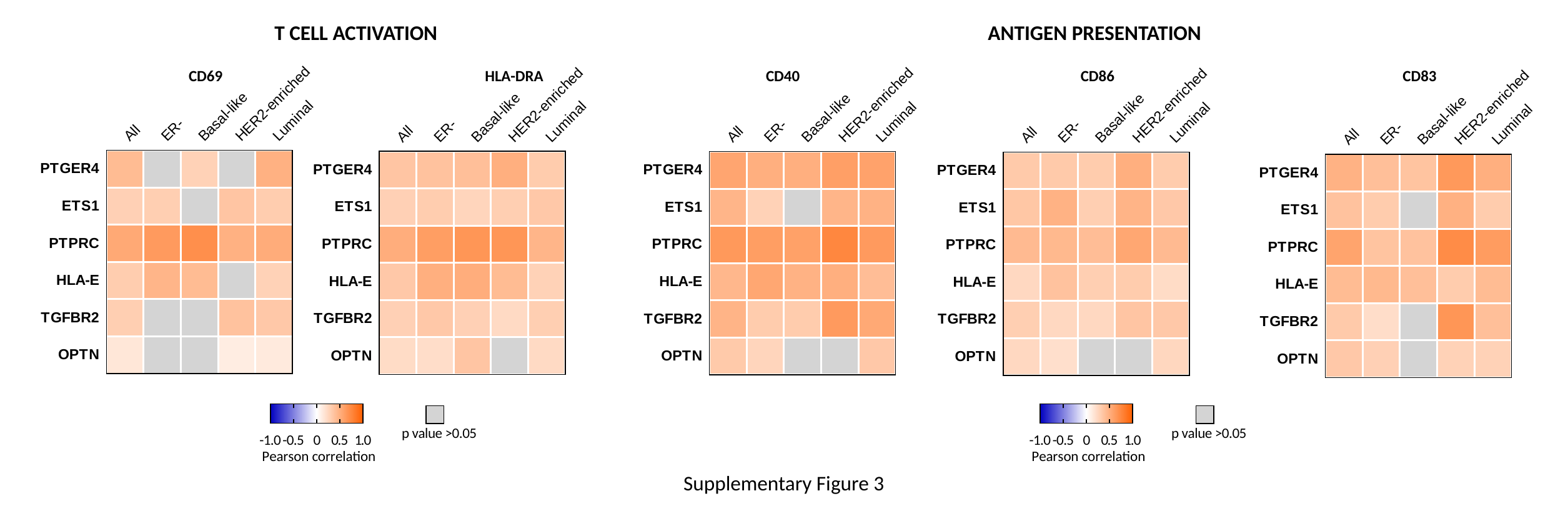

T CELL ACTIVATION
ANTIGEN PRESENTATION
CD69
HLA-DRA
CD40
CD86
CD83
Supplementary Figure 3

Supplement: Supplementary file 1 [file ijms-22-03836-s001.zip › Supplementary Figure 3.pptx]
